# Editorial: Cerebral Palsy: New Developments

**DOI:** 10.3389/fneur.2021.738921

**Published:** 2021-08-11

**Authors:** Antigone Papavasiliou, Hilla Ben-Pazi, Sotiria Mastroyianni, Els Ortibus

**Affiliations:** ^1^Department of Pediatric Neurology, Iaso Children's Hospital, Marousi, Greece; ^2^Department of Pediatric Orthopedics, Assuta Ashdod Hospital, Ashdod, Israel; ^3^Department of Pediatric Neurology, Pan and Aglaia Kyriakou Children's Hospital, Athens, Greece; ^4^Department of Development and Regeneration, KU Leuven, Leuven, Belgium; ^5^Department of Pediatric Neurology, University Hospitals Leuven, Leuven, Belgium

**Keywords:** cerebral palsy, epidemiology, neuroimaging, genetics, development, ethics, muscle

## Definition and Etiology

Cerebral palsy (CP) is defined as a non-progressive permanent disorder of movement and posture attributed to disturbances in the developing fetal and infant brain (1). Diagnosis is based on clinical signs, not on causation. This definition was not modified when brain MRI demonstrated diverse lesions underlying CP phenotypes in 80% of patients (2, 3), nor when the development of a semi-quantitative scale (4) led to studies on CP characteristics, localization, extent, and severity of the brain lesions. Possible amplification and clarification of the definition is under discussion.

Genetic studies revealed that half of CP cases without environmental risk factors have pathogenic genomic deletions or duplications (5) raising questions whether this definition could withstand the discovery of mutations that disrupt brain development and confer risk for CP (6). For example, Beysen et al. reported three patients with spastic CP and genetically confirmed Aicardi-Goutières syndrome. MacLennan et al. argued that similarly to the diagnosis of epilepsy the clinical diagnosis of CP should remain, but prompt appropriate genetic investigation (7). Lewis et al., based on CP genomic data, proposed criteria for CP-associated genes through gene discovery, laboratory research, and clinical application. Horber et al., provided guidance for searching for genetic etiologies based on neuroimaging from the SCPE database. Predominantly white and gray matter injuries (around 50 and 20%, respectively) are considered acquired; genetic factors may increase vulnerability and are considered with positive family history and/or missing causative external factors. In maldevelopments and non-specific/normal findings (around 11% each), monogenic causes are likely. In miscellaneous MRI findings, a possible genetic origin may be considered. Wu F. et al. analyzed the most cited imaging articles in CP over three decades and concluded that multi-modality neuroimaging and high-level evidence-based methodologies could be used in future research to further elucidate pathophysiology, prognosis and efficacy of proposed treatments. Reviews emphasized the need for uniform procedures of MRI classification (8), image acquisition, and common outcome measures (9, 10). A common data language for research, such as the common data elements for CP proposed by the AACPDM, will facilitate comparison between studies (11).

## Epidemiology

CP prevalence remained relatively stable over several decades; fluctuations reflected advanced perinatal and neonatal care. A decrease in the overall prevalence was preceded by reports on decreasing prevalence in low BW children (12–16). Also, severe CP prevalence is declining in preterm and low BW children with severity remaining unchanged in those extremely preterm or with very low BW (12, 13). Tracking the prevalence and clinical features of patients with CP is inherently related to the quality and amount of the collected data (17). Therefore, data from large population-based registries are valuable. Arnaud et al. examined the prevalence and severity of CP in 2,273 preterms, birth-years 2004-2010, from 12 population-based European registries; CP prevalence decreased except for extremely immature children, with the most severely affected showing a similar trend.

Of particular interest are the moderately and late preterm infants (32–34 and 34–36 weeks GA, respectively) representing the majority of all preterms (18) with higher risk than term infants in mortality, poor short-term and long-term outcomes, including impaired motor function (19). Smyrni et al. reported outcomes of a cohort of moderately and late preterm infants (191 out of 1,016 with CP), derived from a population-based registry. Moderately preterm-born with CP were more likely to have a history of N-ICU admission and require respiratory support than late preterm neonates. BW was a strong predictor for early neonatal problems in both groups. The majority in both groups had bilateral spastic CP, white matter lesions and comparable GMFCS levels.

## Functional Deficits

Spastic CP is the prevailing type and the most amenable to treatment. Main neural impairments characterizing spastic CP include spasticity, decreased selective muscle control and poor postural stability. Secondary non-neural musculoskeletal impairments are altered intrinsic muscle structure, muscle contractures, and bony deformities. Muscle weakness is a predominant impairment highly associated with functional disability that depends both on neural activity and on intrinsic muscle structure. In fact, weakness and contractures are significant contributors to motor deficits and progressive disability with growth, characterizing this condition.

Hanssen et al., discussed the disproportional decrease of muscle size and strength around the knee and ankle joints in spastic CP and highlighted the large variability in the contribution of muscle size to muscle weakness. In a cross-sectional study of the development of lower limb strength in 160 ambulatory patients with bilateral spastic CP and 86 typically developing controls, aged 7–16 years, Darras et al., showed that patients exhibited lower strength values in lower limbs than controls, more pronounced in the severely impaired. A pattern of strength imbalance between antagonistic muscle groups was documented in all ages suggesting that strength imbalances are inherent to CP early on and do not develop with age.

De Beukelaer et al. compared medial gastrocnemius characteristics in Hereditary Spastic Paraparesis and Bilateral Spastic CP and found in both significantly smaller muscle volumes compared to controls concluding that treatment in these two conditions does not have to be different.

Altered muscle structure in CP relates to the pathophysiology of muscle contractures. Howard and Herzog review basic science and imaging studies which provide explanations for muscle stiffness and decreased muscle volume and length in CP. Concerns are expressed on the effect of botulinum toxin on muscle histology based on findings from preclinical animal models. It should be noted that no data exist on muscle histopathological alterations following other spasticity treatments such as, physiotherapy, orthoses, Intrathecal Baclofen and Selective Dorsal Rhizotomies.

Cognitive dysfunction is equally challenging as motor impairment but less studied. Intellectual disability epidemiology as related to imaging and clinical phenomenology is systematically monitored in large CP registries (20, 21). Himmelmann et al. examined structure-function relationships based on MRIs from 3,818 patients with CP (birth-years 1999-2009), from 20 European registers. The Impairment Index (22) showed worse associations of bilateral than unilateral compromise with motor impairment, intellectual disability, vision and hearing impairment and epilepsy. Associations between extent and localization of brain lesions and cognitive function are examined but heterogeneity of cognitive findings with similar MRI lesions and the effects of early brain plasticity raise questions as to the utility of this approach. Few studies assessed cognitive profiles or developmental trajectories of cognition in children with CP (23, 24). Intellectual disability is often overestimated. Tests need to be adapted so that cognition can be reliably assessed, especially in very young children and those with severe speech and motor impairments. Visual-spatial abilities, language and executive functions have been reported on, but other determinants of cognition, such as memory, are less studied (24, 25). In bilateral spastic CP uneven cognitive profiles to the advantage of verbal potential are shown; lower performance IQ is attributed to lower visual-spatial reasoning. Visual – perceptual impairment is reported in children with lower cognitive functioning, but also with normal cognition, unrelated to non-verbal cognitive functioning (26, 27).

## New Management Trends

Treatments performed outside a rehabilitation setting gain popularity emphasizing the importance of parent empowerment in caring for their children. Constraint-induced movement therapy in unilateral CP is a program with proven efficacy given in the home environment or during intensive “camp” sessions (28). Wu C-L. et al. demonstrated feasibility of this program in preschool after botulinum toxin injections with improvements in self-care and hand function.

The need for caution in overestimating the abilities of children when tested in controlled environments is discussed. Wiedmann et al., illustrate the feasibility of measuring walking speed in children with CP with 3D accelerometers in real life and show that generally, patients and controls walk slower in a natural environment than in the lab.

During the COVID-19 outbreak remote assessments and treatments proved helpful but underutilized (29–31). The need to set up such initiatives increased (32). Telemedicine became the main communication route between care providers, patients and caregivers. Ben-Pazi et al. describe the accelerated telemedicine impact during the pandemic in CP care in terms of clinical accessibility, continuity of care, prevention, multidisciplinary approach, and participation. Studies need to examine if teleservices can prevent/reduce long-term comorbidities such as hip dislocation.

Technology has changed the scene for the treatment of CP however, some patients with painful syndromes may not be treated with advanced surgical procedures and palliative techniques may be required. Pain affects patients' quality of life and becomes challenging for patients, families, and physicians. Koch et al. presented four palliative options for severely painful spastic hip dislocation, in those with contraindications for reconstructive surgery. Case studies and technique comparisons show how old approaches can become new trends with high acceptability by patients and families.

Technological advances and societal changes create new ethical dilemmas for those involved in the care of patients with CP. Dan reviews principles of clinical bioethics and provides a theoretical approach toward organization of the clinician's thinking process and a constructive dialogue with patients and families.

## Conclusion

CP, the most common cause of childhood disability, attracts researchers from different disciplines. It is a hot topic for neuroscientists as genetic research unveils the contribution of gene mutations to causation and a promising field for the study of brain structural connectivity and plasticity during development or post-intervention. The need to further elucidate pathophysiological mechanisms in order to develop neuroprotective treatments in those at risk for CP remains. Lastly, research on clinical issues, interventions, and outcomes served by many different medical and therapeutic disciplines, creates a unique setting for collaborative and multifaceted work for the purpose of improving quality of life in children with CP and their families. As a result, CP attracts researchers of neurodevelopment, technology experts, as well as from the therapeutic community and remains an “old” but always challenging problem for all.

## Author Contributions

AP contributed to the design of the editorial, analysis and interpretation of the submitted articles, drafting of the editorial, and finalizing. SM, EO, and HB-P revisited the content, added paragraphs and relevant references. All authors read and approved the article.

## Conflict of Interest

The authors declare that the research was conducted in the absence of any commercial or financial relationships that could be construed as a potential conflict of interest.

## Publisher's Note

All claims expressed in this article are solely those of the authors and do not necessarily represent those of their affiliated organizations, or those of the publisher, the editors and the reviewers. Any product that may be evaluated in this article, or claim that may be made by its manufacturer, is not guaranteed or endorsed by the publisher.
